# Injury to the Brachial Plexus Resulting in Gangrene of the Arm and Fore-arm

**Published:** 1873-08

**Authors:** C. C. F. Gay

**Affiliations:** Surgeon to the Buffalo General Hospital


ART. IV.—*Injury to the Brachial plexus resulting in gangrene of the arm and fore-arm—amputation.* By C. C. F. GAY, M. D., Surgeon to the Buffalo General Hospital.

Mrs. M. A., aged 35 years, was attacked by a person who indeavoured to cut her throat with a razor, on February 24th, 1872. By efforts to defend herself, she received a cut with the razor upon the right arm, just above the flexure of the elbow joint, extending across the arm transversely, and deviding the descending branches of the Brachial plexus.

She was attended by her Physician until March 9th, when I was called to visit her. She was sitting up in her chair, supporting the arm upon a pillow which rested upon her lap. On removing the bandages, the hand, fore-arm, and a portion of the arm were found to be gangrenous. Suppuration was copious just above the elbow, and posteriorly, near the axilla.

The patient would not consent to amputation at this morning visit, but in the afternoon yielding assent, I imputated at the shoulder; assisted by Dr. McNeal and Mr. Bartow, the latter compressing the axillary artery with his fingers.

Primary union was obtained, and in three weeks the patient walked to the Court House, to give testimony.

Remarks.—I could not learn from the history of the case, that the brachial artery was wounded, although both median and ulnar nerves were divided.

There was considerable hemorrhage reported at the time the wound was inflicted, but not sufficient to indicate injury to the artery. No hemorrhage occurred after the first dressing, nor was ligation considered necessary by the attending physicians. The death of the fore-arm was simply and alone the result of the division of the nerves.

An intimation was made that damages would be brought against the medical attendant, as the loss of the arm was thought to be owing to too tight bandaging. But the dressings were properly applied and renewed daily, so that there seemed no just grounds for an attempt to mulct the attendant in damages.

During the operation for amputation the axillary artery was so effectually compressed by my assistant that scarcely any hemorrhage occured.

Thirteen days after the injury was inflicted, the life of the patient necessitated amputation. The line of demarcation was distinct above the elbow, and the fore-arm had been doubtless gangrenous two or three days before; reducing the time to ten days from receipt of injury to total loss of all vitality below division of the nerves.

*Epithelioma of eight years duration—Amputation of the left arm at the shoulder.*

Mr. J. S., aged 47 years, residing at West Seneca. I saw this patient October 16th, 1872. Eight years previous to my visit to him, a tumor made its appearance upon the elbow. The disease progressed until two-thirds of the arm and as much of the fore-arm became involved. There had never been much pain present, but the integument had become disintegrated, leaving an ulcerated surface, the offensiveness of which had become intolerable. The ulcerated surface presented an angry and fungated appearance, the edges of which were elevated, irregular and granulated. The discharge was acrid and the skin became eroded wherever this discharge came in cantact with it. The disintegrating process had extended so as to involve a large portion of the anterior aspect of both the arm and fore-arm.

The general health of the patient had but recently given evidence of impairment. I advised amputation and proceeded at once to perform it in the presence of several medical gentlemen.

The subclavian artery was compressed by Dr. Barnes. Amputation was made at the shoulder, leaving just enough tissue of the arm to form a flap. Hemorrhage occurred from four vessels which were ligated. Compression of the subclavian did not sufficiently control the hemorrhage. Compression of the axillary artery would have been more efficient. The stump was dressed and primary union obtained.

The patient immediately improved in health, and gained in flesh until the following January, when he began to cough, expectorating blood occasionally. I visited the patient January 27th, 1873, found him laboring under phthisis pulmonalis of which he died March 2d, 1873.

Remarks.—That Epithelioma is essentially a local affection is evidenced by its duration, and from the other fact as a sequence, that the general system is not invaded or contaminated by the poison until the local affection has existed for a considerable period of time. It may require years to develope general symptoms.

For eight years this man, though laboring under the local disease was comparatively well otherwise. After removal of the diseased arm, and closure of the stump there was not during the remainder of the man's life any pain, tenderness or purulent discharge, yet the system had become infected—a condition that might have been avoided—if radical means had been resorted to at any time during the earlier years of the malady.

